# Scalable Production of Equine Platelet Lysate for Multipotent Mesenchymal Stromal Cell Culture

**DOI:** 10.3389/fbioe.2020.613621

**Published:** 2021-01-21

**Authors:** A. Hagen, H. Lehmann, S. Aurich, N. Bauer, M. Melzer, J. Moellerberndt, V. Patané, C. L. Schnabel, J. Burk

**Affiliations:** ^1^Equine Clinic (Surgery, Orthopedics), Justus-Liebig-University Giessen, Giessen, Germany; ^2^Department of Veterinary Clinical Sciences, Small Animal Clinic, Justus-Liebig-University Giessen, Giessen, Germany; ^3^Institute of Hygiene and Infectious Diseases of Animals, Justus-Liebig-University Giessen, Giessen, Germany; ^4^Department of Veterinary Clinical Sciences, Clinical Pathology and Clinical Pathophysiology, Justus-Liebig-University Giessen, Giessen, Germany; ^5^Faculty of Veterinary Medicine, Institute of Immunology, Leipzig University, Leipzig, Germany

**Keywords:** mesenchymal stromal cells, platelet concentrate, platelet lysate, fetal bovine serum, equine, cell culture

## Abstract

Translation of multipotent mesenchymal stromal cell (MSC)-based therapies is advancing in human and veterinary medicine. One critical issue is the *in vitro* culture of MSC before clinical use. Using fetal bovine serum (FBS) as supplement to the basal medium is still the gold standard for cultivation of many cell types including equine MSC. Alternatives are being explored, with substantial success using platelet lysate-supplemented media for human MSC. However, progress lags behind in the veterinary field. The aim of this study was to establish a scalable protocol for equine platelet lysate (ePL) production and to test the ePL in equine MSC culture. Whole blood was harvested into blood collection bags from 20 healthy horses. After checking sample materials for pathogen contamination, samples from 19 animals were included. Platelet concentrates were prepared using a buffy coat method. Platelets, platelet-derived growth factor BB, and transforming growth factor β1 concentrations were increased in the concentrates compared with whole blood or serum (*p* < 0.05), while white blood cells were reduced (*p* < 0.05). The concentrates were lysed using freeze/thaw cycles, which eliminated the cells while growth factor concentrations were maintained. Donor age negatively correlated with platelet and growth factor concentrations after processing (*p* < 0.05). Finally, all lysates were pooled and the ePL was evaluated as culture medium supplement in comparison with FBS, using adipose-derived MSC from four unrelated donor horses. MSC proliferated well in 10% FBS as well as in 10% ePL. However, using 5 or 2.5% ePL entailed highly inconsistent proliferation or loss of proliferation, with significant differences in generation times and confluencies (*p* < 0.05). MSC expressed the surface antigens CD90, CD44, and CD29, but CD73 and CD105 detection was low in all culture media. Adipogenic and osteogenic differentiation led to similar results in MSC from different culture media. The buffy coat method is useful to produce equine platelet concentrate with increased platelet and reduced white blood cell content in large scales. The ePL obtained supports MSC expansion similar as FBS when used at the same concentration (10%). Further investigations into equine MSC functionality in culture with ePL should follow.

## Introduction

Cell-based therapies are promising tools for regenerative treatment of human and animal diseases. While some approaches have already been successfully implemented in clinical practice, most are still in developmental stages. In horses, particularly treatment of orthopedic conditions with multipotent mesenchymal stromal cells (MSC) has a well-documented history (Smith, 2003; Pacini et al., 2007; Godwin et al., 2012; Renzi et al., 2013; Schauwer et al., 2013; Ferris et al., 2014; Mariñas-Pardo et al., 2018; Broeckx et al., 2019). One current challenge in the development of successful cell-based products, irrespective of the target species, is the implementation of manufacturing processes that comply with legal regulations and maintain cellular potency. As most research focuses on cell therapies for human patients, it remains particularly challenging to adopt suitable procedures for cells derived from large animal species. However, this is crucial with respect to the treatment of companion animals as well as for the use of large animal species in translational studies. Both, clinical application and translational aspects apply to the equine species.

The cell culture medium is a critical element in cell manufacturing processes and may strongly impact on cell quality and efficacy of therapies. To supply the cells with hormones, nutrients, and growth factors, the supplementation of basal medium with fetal bovine serum (FBS) is still the gold standard for *in vitro* culture of many cell types, including equine MSC (Doucet et al., 2005; van der Valk et al., 2010; Bieback, 2013; Burnouf et al., 2016). However, the use of FBS is afflicted with several problems, including an expected shortage of supply (Jayme et al., 1988; Jochems et al., 2002), the ethically critical harvesting procedures (Hodgson, 1995; Jochems et al., 2002; van der Valk et al., 2004), inconsistent quality (Gstraunthaler, 2003; Zheng et al., 2006; Baker, 2016), and its xenogeneic use with the possibility of recipient immune reactions (Sundin et al., 2007; Bieback, 2013) and transmission of bovine pathogens (Erickson et al., 1991; World Health Organization, 2006; Hawkes, 2015). For these reasons, efforts should be made to reduce or replace the use of FBS. In this line, both the European Medicines Agency (EMA) (European Medicines Agency, London, 2013) and the International Society for Cellular Therapy (ISCT) (Karnieli et al., 2017) have recommended the control of quality and safety of FBS and if possible, its replacement. Several alternatives to using FBS have been explored, with most encouraging results using chemically defined/serum-free or platelet lysate (PL)-supplemented media.

For human MSC, commercially available serum-free culture media were developed. However, when investigating the applicability of such serum-free media in equine adipose-derived MSC, we observed differences in morphology and expression of the surface marker CD90, as well as increased aggregation and spontaneous detachment of these MSC (Schubert et al., 2018). These results underlined that culture condition requirements are species-specific with regard to nutrient and growth factor supplementation. Consequently, the use of commercially available serum-free media for large animal MSC is not a consummate approach.

Production and use of human PL (hPL) for MSC cultivation was first reported in 2005 (Doucet et al., 2005). Platelets (PLT) exhibit an important role not only in primary hemostasis but also in wound healing and tissue regeneration. Their α-granules are rich in chemokines and growth factors, such as platelet-derived growth factor (PDGF), basic fibroblast growth factor (bFGF), insulin-like growth factor (IGF), transforming growth factor-β (TGF-β), vascular endothelial growth factor (VEGF), epidermal growth factor (EGF), adhesion factors, and enzymes (Blair and Flaumenhaft, 2009; Schallmoser et al., 2009; Astori et al., 2016; Burnouf et al., 2016). When released upon PLT activation, these factors support cell proliferation and recruitment (Golebiewska and Poole, 2015). Therefore, PL was considered as a suitable acellular culture media supplement. By now, several studies have shown that hPL is better suited for human MSC expansion than FBS (Blande et al., 2009; Mojica-Henshaw et al., 2013; Mohammadi et al., 2016; Becherucci et al., 2018; Schallmoser et al., 2020), and that the MSC cultured with hPL fulfill the definition criteria recommended by the International Society for Cellular Therapy (ISCT) in 2006 (Dominici et al., 2006; Mojica-Henshaw et al., 2013; Becherucci et al., 2018). Interestingly, it was reported that hPL already replaces FBS in 77% of the good manufacturing practice protocols for MSC production in human medicine (Trento et al., 2018), demonstrating significant progress.

In order to follow this development, first studies have already been carried out with equine PL (ePL) for equine MSC cultivation (Del Bue et al., 2007; Seo et al., 2013; Russell and Koch, 2016; Gilbertie et al., 2018; Naskou et al., 2018; Yaneselli et al., 2019). The equine MSC cultured with ePL showed similar proliferation rates, a fibroblast-like morphology, trilineage differentiation, and improved viability compared with FBS-supplemented cultures (Seo et al., 2013; Naskou et al., 2018) and maintained their immunomodulatory properties (Naskou et al., 2018; Yaneselli et al., 2019). Thus, based on the current state of knowledge, ePL is a promising alternative to FBS for the cultivation of equine MSC. However, in contrast to human blood products, availability of ePL is very limited, entailing the necessity of in-house production. So far, published ePL production procedures mainly include platelet-rich-plasma-based methods in small-scale syringe format (Del Bue et al., 2007; Seo et al., 2013; Russell and Koch, 2016; Gilbertie et al., 2018; Yaneselli et al., 2019) and a plateletpheresis-based method (Naskou et al., 2018), which is scalable but requires specialized equipment. Hence, the procedures used by different laboratories comprise highly distinct approaches, limiting the comparability between studies.

The aim of this study was to provide an ePL production protocol which does not rely on specialized equipment, could easily be adopted by other laboratories, and is scalable to production of large batches. For this purpose, we evaluated a procedure based on the most commonly used techniques in human medicine (Burnouf et al., 2016), a buffy coat-based method to obtain PLT concentrate, followed by repeated freeze/thaw cycles for PLT lysis. In a second step, we tested the obtained ePL in comparison with FBS for its suitability for MSC culture.

## Materials and Methods

### Blood Collection

Whole blood for ePL preparation was collected over a period of 2 months from 20 healthy warmblood horses of similar breeds (5 geldings, 14 mares, and 1 stallion) aged 4–15 years (median: 9 years; interquartile range (IQR): 6) after approval by the local regulatory authority (i.e., regional council Giessen, A14/2019). These donor horses had not received any medication in the last 2 weeks as confirmed by their owners. Furthermore, their health status was evaluated by clinical examination and blood tests comprising complete blood counts with ethylenediaminetetraacetic acid (EDTA) whole blood, blood chemistry with Li-heparin blood and serum, as well as microbiological and virological analyses as specified below.

The whole blood was obtained aseptically from the jugular vein. One milliliter lidocaine hydrochloride 2% was administered subcutaneously at the venipuncture site. A permanent venous catheter (12 G) was inserted in cranial direction and fixed with non-resorbable sutures. First, blood samples were collected into tubes for the blood tests specified above, as well as for an erythrocyte sedimentation rate test.

A total of 2 L whole blood was then collected into four 600 ml commercial blood bags loaded with 70 ml citrate-phosphate-dextrose (CPD; Composelect, Fresenius Kabi, Bad Homburg, Germany) (500 ml whole blood in each), which had been connected via a four-way connector (LS-four-way-connector, B. Braun, Melsungen, Germany) using a sterile tube welder (Compodock, Fresenius Kabi). A blood donation scale (MW5001 electronic, Biotrans GmbH, Dreieich, Germany) was used to standardize the filling volume of the blood bags. After the blood collection was completed, the samples were placed in a CompoCool®Box (Fresenius Kabi) containing butane-1,4-diol cooling plates to cool the whole blood to 20°C within a short time and to improve temperature uniformity. The blood was left there for at least 2 h and a maximum of 3 h until processing in the laboratory. Three of the four blood bag samples per horse were processed as described in the following.

### Platelet Concentrate and Lysate Preparation

**Figure 1:**
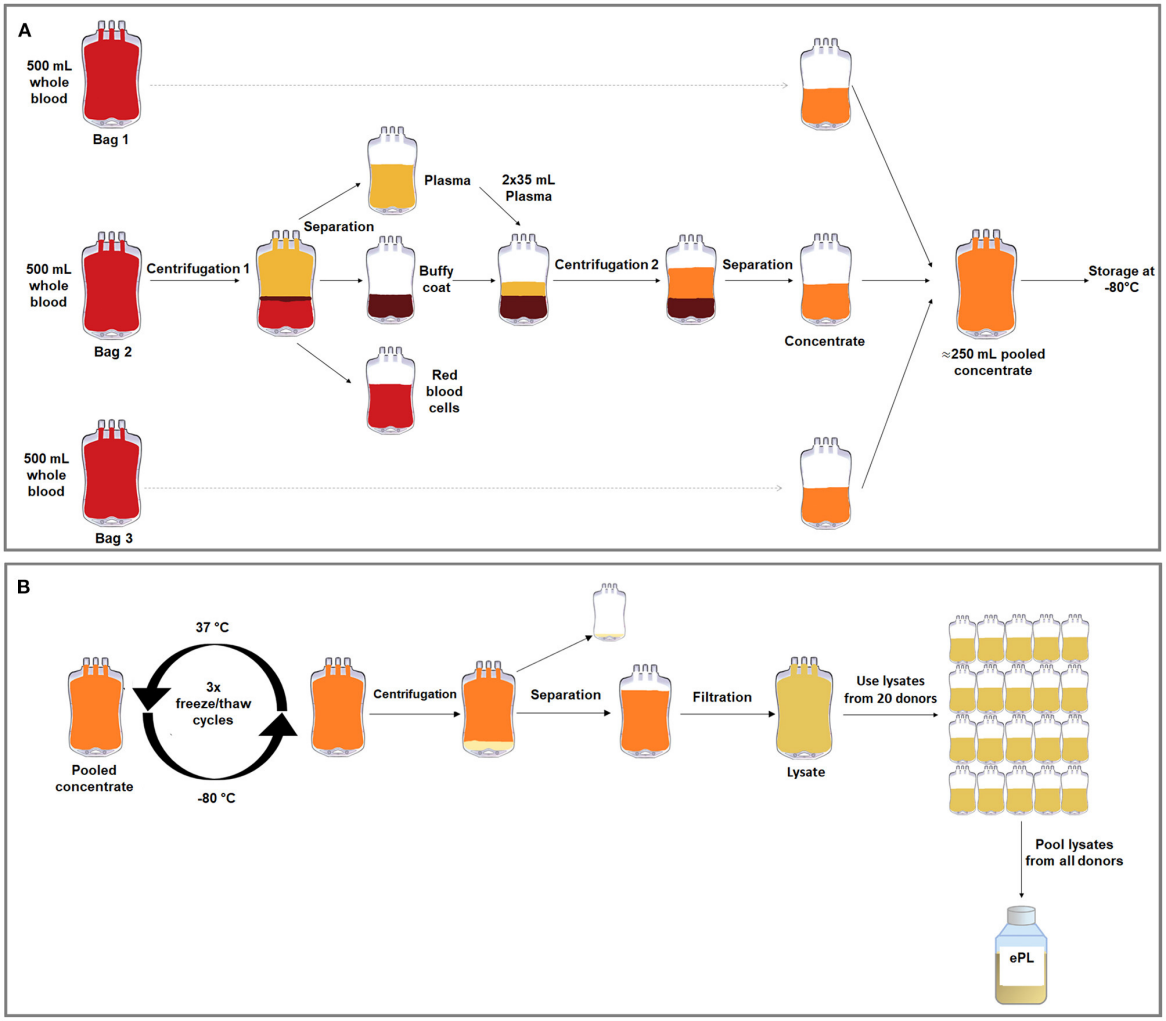
Graphical overviews of the buffy coat-based method to produce concentrate from equine whole blood in blood collection bags **(A)** and the freeze/thaw cycle-based lysis method to produce ePL from this concentrate **(B)**.

The blood bags were centrifuged at 711 × *g* for 20 min using a commercial centrifuge designed for blood separation (Hettich Rotanta 460R, Andreas Hettich GmbH & Co.KG, Tuttlingen, Germany) with acceleration settings of 1, deceleration settings of 0, at 22°C, for blood component separation. The buffy coat was recovered by separating the plasma, and the erythrocyte concentrate by a top-bottom method with a blood-separating device (Optipress® II; Fenwal, Baxter S.A., Maurepas, France). The buffy coat was left to rest for 1 h, then 70 ml plasma were added again for resuspension. The resuspended buffy coat was then centrifuged at 159 × *g* for 10 min, acceleration 1, deceleration 0, at 22°C, in the same centrifuge. After that, the resulting supernatant, corresponding to the concentrate, was separated using a manual press (NPBI Holland) (Figure 1).

The concentrates from the three separate blood collection bags from each horse were then pooled in transfer bags (R6R2021, Compoflex, Fresenius Kabi) to obtain one bag of concentrate per horse, referred to as “concentrate pooled,” and frozen at −80°C. In order to lyse the PLT, three freeze/thaw cycles were performed, in which the PLT concentrate was thawed at 37°C in a dry heating device designed to thaw frozen products intended for infusion under continuous agitation (Plasmatherm, Barkey GmbH &CO.KG, Leopoldshoehe, Germany) for 4 h and then frozen again at −80°C for 20 h. Afterwards, the bags were centrifuged at 4,000 × *g* for 30 min, acceleration 9, deceleration 2, in the same centrifuge described above. The supernatant, corresponding to the lysate, was filtered *via* gravity using Macopharma Plas4 filters (Lot 11290588BM, Macopharma, Langen, Germany) for cell debris removal. The PLT lysate from each donor was stored at −80°C, until the lysate units from all suitable horses (*n* = 19) were pooled under aseptic conditions (Figure 1). The lysate from the remaining horse was not included due to a positive herpes virus finding.

### Microbiological and Virological Assessment

Absence of pathogens was confirmed in blood samples from the individual animals as well as in the final pooled ePL before its use in cell culture. This comprised a bacteriological analysis with Oxoid signal blood culture system (BC0100M, Oxoid Limited, Hampshire, England), incubated at 37°C for 7 days. On days 1, 2, 4, and 6, the cultured blood was streaked on blood agar (Blood Agar Base, Oxoid, Wesel, Germany) containing 5% defibrinated sheep blood and on water-blue metachrome-yellow lactose agar (Water-blue Metachrome-yellow Lactose Agar acc. to Gassner, sifin diagnostics, Berlin, Germany). The plates were incubated under aerobic conditions at 37°C for 48 h. Additionally, Brain-Heart-Infusion agar (Brain Heart Infusion Agar, Oxoid) was incubated under microaerobic conditions (10% CO_2_, 37°C) and analyzed after 24 and 48 h. Schaedler agar (BBL™ Schaedler Agar, Becton Dickinson GmbH, Heidelberg, Germany) and Columbia agar (Columbia Agar (base), E. Merck, Darmstadt, Germany) were incubated at 37°C for 72 h under anaerobic conditions in a jar using AnaeroGen™ gas sachets (AnaeroGen™ 2.5L, Oxoid). For selective culturing of fungi, Kimmig agar (Agar for fungi (base) acc. to Kimmig modified, E. Merck) was incubated for 72 h at 28°C under aerobic conditions. Lastly, a 16S ribosomal RNA gene polymerase chain reaction analysis for mycoplamas (van Kuppeveld et al., 1992) was performed. In addition, a virological examination for equine herpes virus 1 and 4 (virus genome detection), equine arteritis virus (antibody detection by serum neutralization test), equine infectious anemia virus (antibody detection), and virus cultivation in cell culture was commissioned.

### Platelet and Leukocyte Counts

Complete blood counts were obtained from samples harvested at different processing stages using an automated flow cytometric hematology analyzer (ADVIA 2120i, Siemens Healthcare GmbH, Erlangen, Germany) with Multispecies software MS 5.9. This included EDTA blood samples, citrated whole blood samples from each blood collection bag before further processing, plasma samples from each collection bag, concentrate samples from each collection bag, pooled concentrate from each animal, lysate from each animal, and the final ePL pooled from all horses.

### Growth Factor Quantification and Chemical Analyses

Growth factor concentrations were analyzed in serum, plasma, pooled concentrate, and lysate from each horse, as well as in the final pooled ePL. All samples including the concentrates had been stored at −80°C before the growth factors were measured. For comparison, the same growth factors were also measured in the FBS which was used as standard cell culture medium supplement. Specifically, platelet-derived growth factor (PDGF-BB) and transforming growth factor beta 1 (TGF-β1) were quantified using the respective Quantikine ELISA kits (catalog numbers #DBB00 and #DB100B, R&D Systems, Minneapolis, MN, USA), which have been used for equine samples in previous studies (Anderson et al., 1998; Desjardins et al., 2004; Donnelly et al., 2006; Boswell et al., 2014; McClain and McCarrel, 2019). Procedures were performed according to the product manuals, which included TGF-β1 activation with hydrochloric acid, and samples were analyzed using an Infinite F50 (Tecan) plate reader and the corresponding Magellan software (Tecan Ltd., Maennedorf, Switzerland).

Furthermore, in the same samples, according to the chemical quality analysis of FBS, electrolyte and total protein analyses were performed using a blood gas and electrolyte analyzer (Cobas b 123 POC system, Roche Diagnostics GmbH, Mannheim, Germany). The total protein and albumin content was determined using a clinical chemistry analyzer C400 (Pentra C400 Option I.S.E, HORIBA ABX SAS, Montpellier, France).

### MSC Culture With FBS and ePL Media Supplements

The final pooled ePL was evaluated as cell culture supplement for equine adipose-derived MSC, in comparison with FBS. Adipose-derived MSC had been harvested from four healthy standard-bred horses (three geldings, one stallion) aged 5–9 years (median: 8 years; IQR: 1) in the framework of an unrelated previous study as approved by the respective local authority (Landesdirektion Leipzig, TV34/13). MSC had been isolated by collagenase digestion and expanded in FBS-supplemented culture medium until cryopreservation. The cells were thawed and seeded in Dulbecco's modified Eagle's medium (1 g/L glucose; Gibco®, ThermoFisher Scientific, Darmstadt, Germany) supplemented with either 10% FBS (Lot: 2078409, Gibco®, ThermoFisher Scientific) or 10, 5, and 2.5% ePL, 1% penicillin-streptomycin, and 0.1% gentamycin. When using ePL, 1 U/ml heparin-natrium (B. Braun, Melsungen, Germany) was additionally added to the culture medium. MSC were then cultured under standard conditions (humidified atmosphere, 37°C, 5% CO_2_) for at least one passage prior to any experiment to allow for possible adaptations to the respective culture media. All experiments were performed using the MSC from all donor horses and except for the flow cytometry experiments, mean values from technical replicates were used for further statistical analyses.

### Cell Proliferation and Viability Assays

For estimation of the generation time, population doublings in passages 2 and 3 were evaluated. MSC were seeded at a density of 3,000 cells/cm^2^ and incubated for 5 days, with a medium change after 3 days. Phase-contrast photomicrographs were obtained at standardized settings using a Nikon Eclipse Ts2-FL microscope with a DS-Fi3 camera (Nikon GmbH, Duesseldorf, Germany) at day 5 before passaging. Cells were then trypsinized and counted using a hemocytometer and trypan blue for exclusion of dead cells. The generation times were calculated using the following formula:

$$\begin{array}{l} \text{Generation time}=\frac{\text{days in culture}}{\frac{\ln\left(\frac{\text{cell count harvest}}{\text{cell count seeding}}\right)}{\text{ln}2}} \end{array}$$

Using Fiji ImageJ software, the images obtained before passaging were uniformly enhanced in contrast, a background subtraction was done and images were binarized, with adapted thresholds for each image, and the confluent area within each image was measured. In addition, MSC metabolic activity was measured at day 1 and 5 using a tetrazolium compound (MTS) assay according to the manufacturer's instructions (CellTiter 96® AQueous One Solution Cell Proliferation Assay, Promega, Mannheim, Germany). The mean absorbance at day 5 was divided by the mean absorbance at day 1 as an indicator for metabolic activity.

### Immunophenotyping

Immunophenotypic analyses were performed in passage 3 MSC to assess the presence of inclusion and exclusion marker antigens recommended for MSC characterization, based on procedures established previously for equine MSC (Paebst et al., 2014). Briefly, MSC were analyzed by flow cytometry for CD29, CD44, CD73, CD90, and CD105, as well as for CD14, CD34, CD45, CD79α, and MHC-II. First, MSC were detached in ice-cold 0.01 M EDTA using cell scrapers to prevent loss of surface molecules due to enzymatic detachment. Next, cells were successively incubated with fixable viability dye (1:1,000; eBioscience™ Fixable Viability Dye eFluor™ 780, ThermoFisher Scientific) for 20 min, the respective blocking sera (15% goat serum for CD73 staining and 5% mouse or rat serum for all other stainings, all Sigma-Aldrich, Taufkirchen, Germany), followed by the antibodies detailed in Table 1. Incubation steps were performed protected from light for 15 min at 4°C, with interjacent washing steps in staining buffer (phosphate-buffered saline (PBS) supplemented with 0.01% sodium azide and 10% FBS). Finally, all cells were fixed in 2% paraformaldehyde, washed with PBS, and stored in staining buffer overnight at 4°C. For CD79α staining, fixation and permeabilization solutions (BD Cytofix/Cytoperm™, BD Biosciences, Franklin Lakes, NJ) were used according to the manufacturer's instructions. Flow cytometric measurements of a minimum of 20,000 events per sample were performed on an LSR Fortessa II (BD) equipped with FACS Diva 6.2 software (BD). Data was analyzed using FlowJo™ v10.7 software (FlowJo, LLC, BD Biosciences, Ashland, OR, USA). Live MSC were gated as large cells after duplet exclusion and marker expression gates were set based on the corresponding isotype-, secondary antibody- or conjugate controls.

Table 1Antibodies used for immunophenotyping.

**Table:** 

**Subset**	**Antibody**	**Host species, isotype**	**Clone**	**Reactivity**	**Company**	**References**	**Dilution**
I	CD29-A488	Mouse IgG1	TS2/16	Anti-human	Biolegend, San Diego, CA	Schauwer et al. (2012) and Paebst et al. (2014)	1:20
I	CD44-APC	Rat IgG2b	IM7	Anti-mouse	BD, Franklin Lakes, NJ	Schauwer et al. (2012) and Paebst et al. (2014)	1:100
IV	CD73	Mouse IgG1	10f1	Anti-human	Abcam, Cambridge, UK	Schauwer et al. (2012) and Paebst et al. (2014)	1:5
III	CD90-APC	Mouse IgG1	5E10	Anti-human	BD, Franklin Lakes, NJ	Hillmann et al. (2016)	1:100
II	CD105-PE	Mouse IgG1	SN6	Anti-human	Bio-Rad, Hercules, CA/Serotec, Kidlington, UK	Schauwer et al. (2012) and Paebst et al. (2014)	1:10
II	CD14-APC	Mouse IgG1	134620	Anti-human	R&D, Minneapolis, MN	Braun et al. (2010) and Paebst et al. (2014)	1:50
V	CD14 biotinylated	Mouse IgG1	105	Anti-equine	Cornell University, Dr. Bettina Wagner	Kabithe et al. (2010)	1:1,500
III	CD34-FITC	Mouse IgG3	43A1	Anti-human	AdipoGen Lifesciences, San Diego, CA	Paebst et al. (2014)	1:25
II	CD45-A488	Mouse IgG2a	F10-89-4	Anti-human	Bio-Rad, Hercules, CA/Serotec, Kidlington, UK	Schauwer et al. (2012) and Paebst et al. (2014)	1:5
VI	CD79α-PE	Mouse IgG1	HM57	Anti-human	Bio-Rad, Hercules, CA/Serotec, Kidlington, UK	Schauwer et al. (2012) and Paebst et al. (2014)	1:50
I	MHCII (equine), PE	Mouse IgG1	CVS20	Anti-equine	Bio-Rad, Hercules, CA/Serotec, Kidlington, UK	Kydd et al. (1994) and Lunn et al. (1998), Schauwer et al. (2012), and Paebst et al. (2014)	1:25
IV	Goat anti-mouse Ig3, FITC	Polyclonal IgG		Anti-mouse	Santa Cruz Biotechnology, Dallas, Texas		1:100
V	SAV-APC	APC streptavidin			Biolegend, San Diego, CA		1:250

**Table:** 

**Isotyp control**	**Corresponding antibody**	**Host species**	**Company**	**Dilution**
IgG1κ, A488	CD29	Mouse	Biolegend, San Diego, CA	1:20
IgG2bκ, APC	CD44	Rat	Biolegend, San Diego, CA	1:100
IgG2aκ, APC	CD90	Mouse	Biolegend, San Diego, CA	1:100
IgG1κ, PE	CD105	Mouse	Biolegend, San Diego, CA	1:10
IgG1κ, APC	CD14 (Clone:134620)	Mouse	Biolegend, San Diego, CA	1:50
IgG3κ, FITC	CD34	Mouse	Biolegend, San Diego, CA	1:25
IgG2aκ, A488	CD45	Mouse	Biolegend, San Diego, CA	1:5
IgG1κ, PE	CD79α	Mouse	Biolegend, San Diego, CA	1:50
IgG1κ, PE	MHCII	Mouse	Biolegend, San Diego, CA	1:50

### Trilineage Differentiation

The *in vitro* differentiation assays were performed in passage 2 MSC cultured in 10% FBS, 10% ePL, or 2.5% ePL. The MSC differentiation assay was not performed with 5% ePL, as previous approaches had already shown a tendency for a higher ePL concentration to be more potent and therefore the differentiation was only conducted with one low ePL concentration (2.5%).

For adipogenic differentiation, MSCs were seeded at 1,500 cells/cm^2^ and incubated for 3 days under standard culture conditions. Then, standard medium was replaced by StemPro™ adipogenic differentiation medium (catalog number A1007001, Gibco®, ThermoFisher Scientific) with 0.1% gentamycin and 5% rabbit serum. After 7 days of incubation, samples were fixed with 50% ethanol for 20 min and stained with Oil Red O and hematoxylin counterstain. The intensity of adipogenic differentiation was assessed by two blinded observers using a scoring system based on the percentage of differentiated cells and the size and arrangement of lipid droplets in these cells, as previously described (Gittel et al., 2013).

For osteogenic differentiation, MSC were seeded at 1,000 cells/cm^2^ and incubated for 3 days under standard culture conditions. Then, standard medium was replaced by StemPro™ osteogenic differentiation medium (catalog number A1007201, Gibco®, ThermoFisher Scientific) with 0.1% gentamycin. This medium was changed twice weekly for 21 days of incubation. Cells were fixed with 4% paraformaldehyde for 10 min and von Kossa staining was performed to detect extracellular mineralization. Bright field photomicrographs were obtained, and mean grayscale values were extracted using Fiji ImageJ software.

Chondrogenic differentiation of MSC was performed in pellet culture with 500,000 cells per pellet. MSC were washed in PBS before StemPro™ chondrogenic differentiation medium (catalog number A1007101, Gibco®, ThermoFisher Scientific), and 0.1% gentamycin were added, and then centrifuged at 280 × *g* at 4°C for 5 min to form a cell pellet. Medium was changed twice a week until day 21. Pellets were fixed with 4% paraformaldehyde for 12 h, paraffin sections prepared and stained with Alcian blue and Masson's Trichrome. Samples were evaluated by two blinded observers, based on the Grogan score (Grogan et al., 2006) adapted to the stainings used in the current study (Supplementary File 1). In addition, bright field photomicrographs were analyzed using Fiji ImageJ software (Ruifrok and Johnston, 2001). This included color deconvolution and binarization of the resulting images, followed by the measurement of the percentage areas stained with the respective staining component. Results are presented as ratios of cartilaginous matrix staining and counterstaining (i.e., turquoise to purple staining for Alcian blue and blueish to red staining for Masson's trichrome).

### Statistical Analysis

Statistical analyses and graphical presentation of data were performed using IBM SPSS Statistics 26 and GraphPad Prism 8.4.3. Data obtained during blood processing (*n* = 19) are presented as median and 95% confidence interval. These data were additionally categorized based on donor age [4–9 years (*n* = 11) and 10–15 years (*n* = 8)] and sex [female (*n* = 13) and male (*n* = 6)] for comparisons between these groups. Data from the cell culture experiments (*n* = 4) are presented as scatter plots with median and interquartile range. Correlation between parameters was evaluated based on Spearman's rank correlation, and comparisons between related samples were performed using non-parametric tests for paired samples (Friedman tests with Wilcoxon *post-hoc* tests and Bonferroni correction for multiple testing). For age and sex group comparisons, Mann-Whitney *U*-tests were computed. Differences were considered significant at *p* < 0.05.

## Results

### Platelet Lysate Preparation

#### Absence of Pathogen Contamination

No microbiological contamination was evident in the blood samples from the donors or in the final pooled ePL. However, one donor animal tested positive for herpes viridae. Consequently, the ePL obtained from this horse was not included in the final product and data obtained were excluded from all analyses.

#### Platelet Concentration and WBC Removal

**Figure 2:**
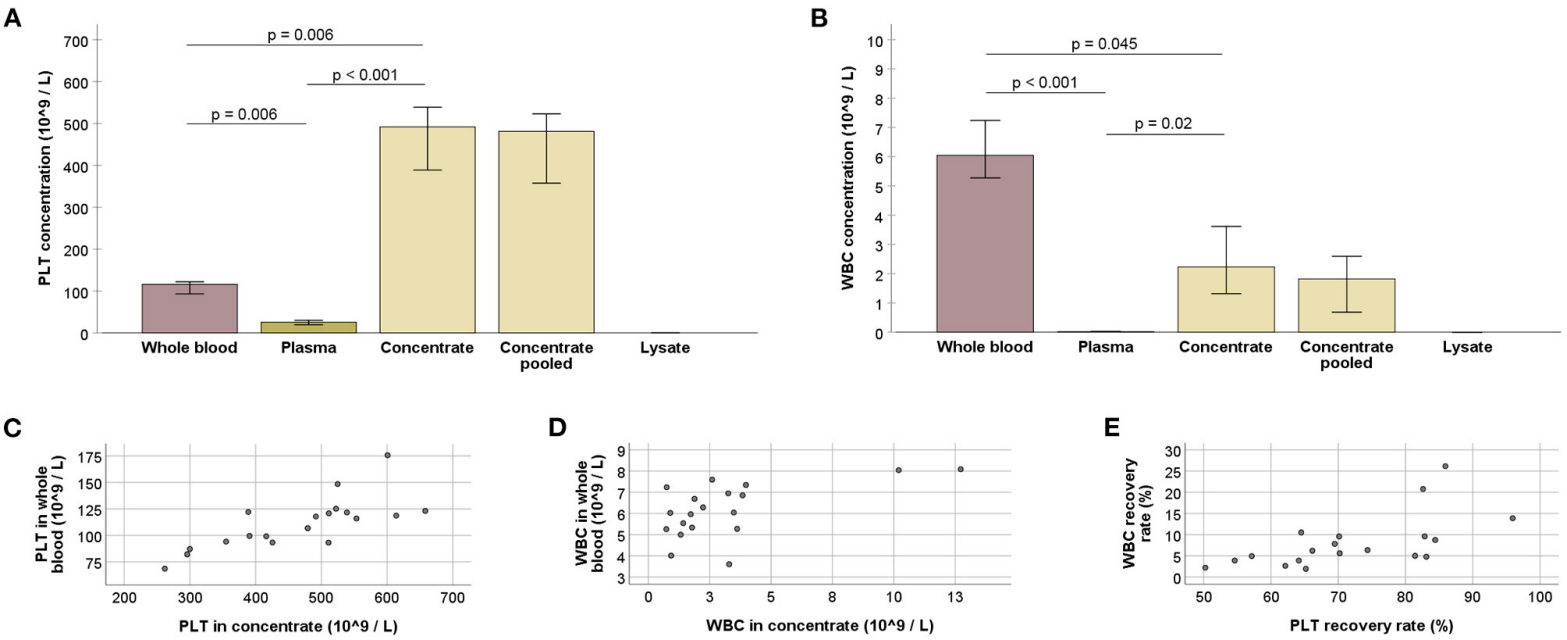
**(A,B)** Platelet (PLT; **A**) and white blood cell (WBC; **B**) counts at the different stages of blood processing; values for whole blood, plasma, and concentrate were obtained using three technical replicates (blood bags), for each horse; values for concentrate pooled and lysate were obtained after all bags from one animal had been pooled; Friedman and Wilcoxon tests for group comparisons were performed for the former (*p*-values are indicated); bars represent the median values, error bars the 95% confidence intervals. **(C–E)** PLT **(C)** and WBC **(D)** counts in whole blood vs. concentrate, and WBC vs. PLT recovery rates **(E)** (*p* < 0.05, based on Spearman's rank correlation). All data were obtained from *n* = 19 horses.

A medium whole blood volume of 504.9 ml (IQR: 25.7) yielded a medium concentrate volume of 86.2 ml (IQR: 7.1) per collected blood bag, with recovery rates of 70.2% (IQR: 19.3) of the total processed PLT and 6.3% (IQR: 2.6) of the total processed WBC. As compared with the whole blood, the obtained PLT concentrates had 4.2-fold increased PLT concentrations and 0.4-fold decreased WBC concentrations (*p* < 0.01 and *p* < 0.05, respectively). The remaining plasma showed low PLT concentrations and very low WBC concentrations (*p* < 0.01 compared with whole blood and concentrate). After lysis of the concentrates, PLT and WBC counts in the lysates were neglectable (Figure 2).

Whole blood and concentrate PLT concentrations correlated strongly (*p* < 0.001 and *r* = 0.743). A moderate correlation was also observed between the respective WBC concentrations (*p* < 0.05 and *r* = 0.564). Furthermore, recovery rates of PLT and WBC were correlated (*p* < 0.01 and *r* = 0.617) (Figure 2).

#### Growth Factor Concentration and Chemical Analyses

**Figure 3:**
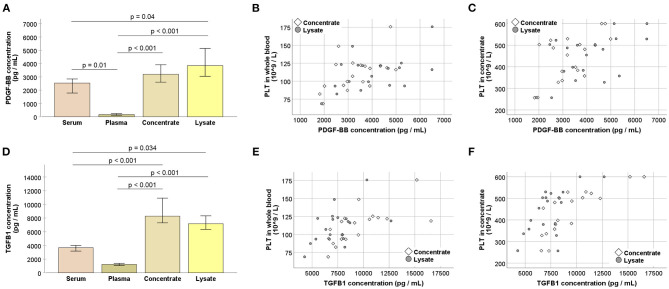
**(A,D)** Platelet-derived growth factor (PDGF-BB; **A**) and transforming growth factor (TGFB1; **B**) concentrations at the different stages of blood processing; serum samples were directly drawn from each horse and the remaining values were obtained after pooling the replicate samples, i.e., blood bags, for each horse; samples were stored at −80°C before analysis; Friedman and Wilcoxon tests for group comparisons were performed (*p*-values are indicated); bars represent the median values, error bars the 95% confidence intervals. **(B–F)** Correlations between whole blood **(B,E)** or concentrate **(C,F)** platelet (PLT) counts and PDGF-BB **(B,C)** or TGFB1 **(E,F)** concentrations in concentrates as well as lysates (*p* < 0.05, based on Spearman's rank correlation). All data were obtained from *n* = 19 horses.

After storage at −80°C, growth factor concentrations were higher in the PLT concentrates (*p* > 0.05 for PDGF-BB and *p* < 0.01 for TGF-β1) as well as in the lysates (*p* < 0.01 for PDGF-BB and *p* < 0.05 for TGF-β1), as compared with the corresponding serum. The plasma showed very low growth factor concentrations (*p* ≤ 0.01 for all comparisons, except for serum vs. plasma TGF-β1) (Figure 3), corresponding to its low PLT content.

Platelet concentrations in the concentrates showed moderate to strong correlations with the growth factor concentrations in the concentrates (*p* < 0.01 and *r* = 0.599 for PDGF-BB; *p* < 0.001 and *r* = 0.785 for TGF-β1) as well as in the lysates (*p* < 0.05 and *r* = 0.526 for PDGF-BB; *p* < 0.01 and *r* = 0.626 for TGF-β1). This same trend was observed for PLT concentrations in whole blood which also correlated with the growth factor concentrations in the concentrates (*p* < 0.05 and *r* = 0.542 for PDGF-BB; *p* = 0.001 and *r* = 0.687 for TGF-β1) and in the lysates (*p* < 0.05 and *r* = 0.514 for PDGF-BB; *p* < 0.05 and *r* = 0.503 for TGF-β1) (Figure 3).

The chemical analysis of the samples from the different production steps showed a stable pH value. Electrolyte, glucose, total protein, and albumin concentrations differed between serum and the samples from lysate production as anticipated due to binding and/or dilution by the anticoagulant citrate-phosphate-dextrose used in the latter. No major changes were observed throughout blood processing. Compared with FBS, glucose and protein concentrations were higher in serum and all steps of lysate production than in FBS, while potassium and lactate concentrations were lower (Table 2).

**Table 2:** pH and electrolyte, glucose, lactate, total protein, and albumin concentrations at the different stages of blood processing; values for serum, plasma, concentrate, and lysate are presented as mean ± SD (*n* = 19).

**Sample**	**pH**	**Na^**+**^ (mmol/L)**	**K^**+**^ (mmol/L)**	**Ca^**2+**^ (mmol/L)**	**Cl^**−**^ (mmol/L)**	${\text{HCO}}_{3}^{-}$ **(mmol/L)**	**Glucose (mmol/L)**	**Lactate (mmol/L)**	**Total protein (g/L)**	**Albumin (g/L)**
Serum	7.54 ± 0.03	141.38 ± 2.01	4.26 ± 0.49	1.79 ± 0.07	105.31 ± 2.53	28.06 ± 1.39	4.63 ± 0.49	2.09 ± 0.39	59.69 ± 2.25	32.26 ± 1.44
Plasma	7.53 ± 0.03	148.71 ± 1.79	3.16 ± 0.25	<0.10	84.04 ± 2.64	19.08 ± 1.22	23.92 ± 1.23	1.55 ± 0.24	51.66 ± 1.83	27.33 ± 1.18
Concentrate	7.47 ± 0.04	147.88 ± 1.54	3.62 ± 0.29	<0.10	84.57 ± 1.73	18.44 ± 1.63	22.62 ± 1.37	2.70 ± 0.63	53.39 ± 1.75	28.11 ± 1.10
Lysate	7.50 ± 0.05	148.00 ± 1.46	3.69 ± 0.30	<0.10	84.74 ± 1.68	17.06 ± 1.19	23.01 ± 1.30	2.87 ± 0.66	53.57 ± 2.08	27.99 ± 1.10
Lysate pooled (ePL)	7.52	147.80	3.68	<0.10	84.50	16.7	23.10	2.90	54.10	27.90
Fetal bovine serum	7.45	138.60	11.21	1.27	106.60	12.7	2.20	17.70	36.80	23.00

#### Donor-Related Parameters Influencing Outcome

**Figure 4:**
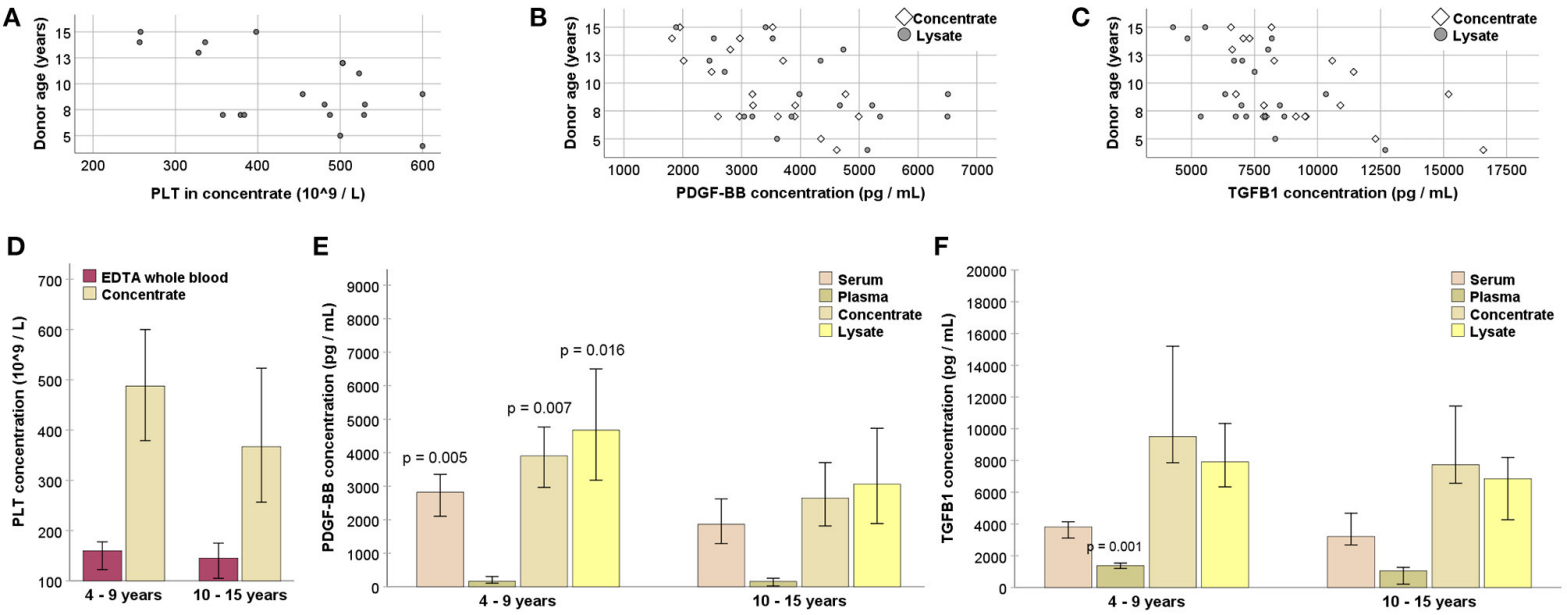
**(A–C)** Negative correlation of donor age with platelet (PLT; **A**), platelet-derived growth factor (PDGF-BB; **B**) and transforming growth factor (TGFB1; **C**) concentrations (*p* < 0.05 based on Spearman's rank correlation). **(D–F)** PLT **(D)**, PDGF-BB **(E)**, and TGFB1 **(F)** concentrations in EDTA whole blood or serum samples drawn directly from the donor horses, as well as in samples from different processing stages (replicate blood bags pooled for each animal), obtained from younger and older donors; pairwise comparisons between the samples from younger and older animals were performed (Mann-Whitney *U*-test; *p*-values are given for significant differences compared with the respective samples from the older age group). Data were obtained from *n* = 11 younger and *n* = 8 older horses.

Donor age was linked to the outcome of blood processing, despite a lack of correlation between age and whole blood PLT or serum growth factor concentrations (*p* > 0.05). Nevertheless, age was negatively correlated with PLT concentrations in the concentrates (*p* < 0.01 and *r* = −0.582), as well as with PDGF-BB and TGF-β1 concentrations in the concentrates (*p* < 0.01 and *r* = −0.627 for PDGF-BB; *p* < 0.05 and *r* = −0.545 for TGF-β1) and lysates (*p* < 0.05 and *r* = −0.483 for PDGF-BB; *p* < 0.05 and *r* = −0.566 for TGF-β1). When grouping the donors based on their age (4–9 vs. 10–15 years), the younger animals had higher serum PDGF-BB concentrations as well as higher PDGF-BB concentrations in the concentrates and lysates (*p* < 0.05). Fewer differences were found for PLT or TGF-β1 concentrations between age groups (Figure 4).

No differences were observed with respect to donor sex (*p* > 0.05 in sex group comparisons for all parameters).

Attempting to identify further attributes of suitable donor animals, parameters typically obtained by routine blood tests were also considered. EDTA whole blood PLT concentrations, similar as the respective values obtained from the blood collection bags, moderately correlated with concentrate PLT concentrations (*p* = 00.01 and *r* = 0.573), but not with the PLT recovery rate. The erythrocyte sedimentation time (median: 37 mm/30 min; IQR: 33.8) appeared to have no predictive effect at all, with no correlation observed for any relevant outcome parameter (*p* > 0.05).

### Platelet Lysate in Equine MSC Culture

#### Growth Factors in Medium Supplements

The pooled ePL used for the cell culture experiments had a PDGF-BB concentration of 3,783 pg/ml, whereas FBS displayed a PDGF-BB concentration near or below the detection level of the assay. The concentration of TGF-β1 was 3,966 pg/ml in ePL and 3,380 pg/ml in FBS.

#### MSC Morphology, Proliferation, and Viability

MSC displayed the characteristic fibroblast-like morphology in all media, yet their shape tended to be more rounded in ePL-supplemented media and MSC supplemented with 10% ePL appeared to grow in more dense clusters.

**Figure 5:**
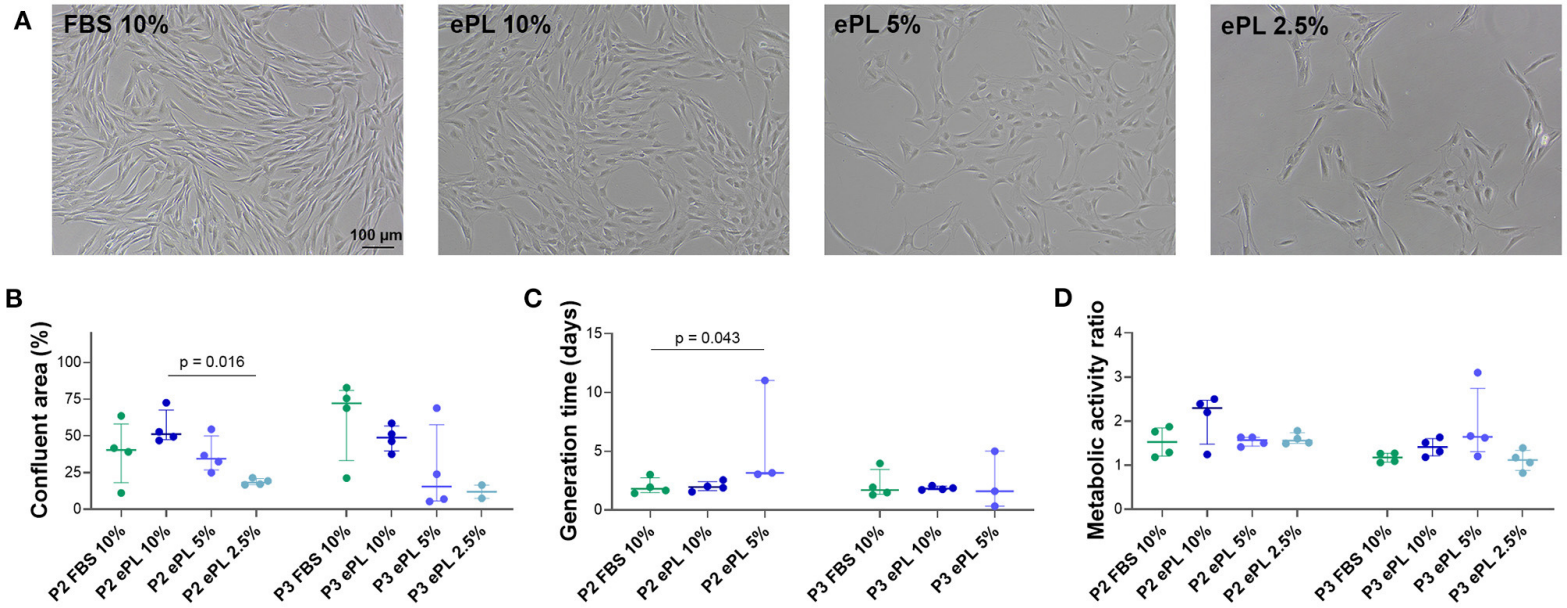
**(A)** Representative phase-contrast photomicrographs of equine mesenchymal stromal cells (MSC) from one donor at day 5 after seeding for population doubling assays in media supplemented with fetal bovine serum (FBS) or equine platelet lysate (ePL). **(B–D)** Confluent area at day 5, measured using Fiji ImageJ **(B)**, generation times calculated from cell doubling numbers **(C)**, and metabolic activity as determined by MTS tetrazolium-based cell proliferation assay **(D)** for MSC in passage 2 (P2) cultured in the different media; Friedman and Wilcoxon tests for group comparisons were performed (*p*-values are indicated); the plots display the individual values, median, and interquartile ranges. Data were obtained from MSC from *n* = 4 donors. Note that missing generation time data are due to the lack of proliferation in these samples (1 out of 4 in the 5% ePL group, 4 out of 4 in the 2.5% ePL group).

The most consistent viability and proliferation was observed in medium supplemented with 10% ePL, but confluency at day 5, generation time, and metabolic activity were similar between 10% ePL MSC and 10% FBS MSC. However, proliferation was highly variable in medium supplemented with 5% ePL and insufficient in medium supplemented with 2.5% ePL. Correspondingly, confluency was lowest in 2.5% ePL medium (*p* < 0.05 compared with 10% ePL in passage 2), and generation time was higher in 5% ePL medium compared with FBS medium (*p* < 0.05 in passage 2) (Figure 5). Here, it is of note that in one out of four samples in 5% ePL and in all four samples in 2.5% ePL, generation time calculation generated negative results, thus these values had to be excluded from further comparative analyses. Yet interestingly, there were no significant differences between groups with respect to MSC metabolic activity (Figure 5).

#### MSC Immunophenotype

**Figure 6:**
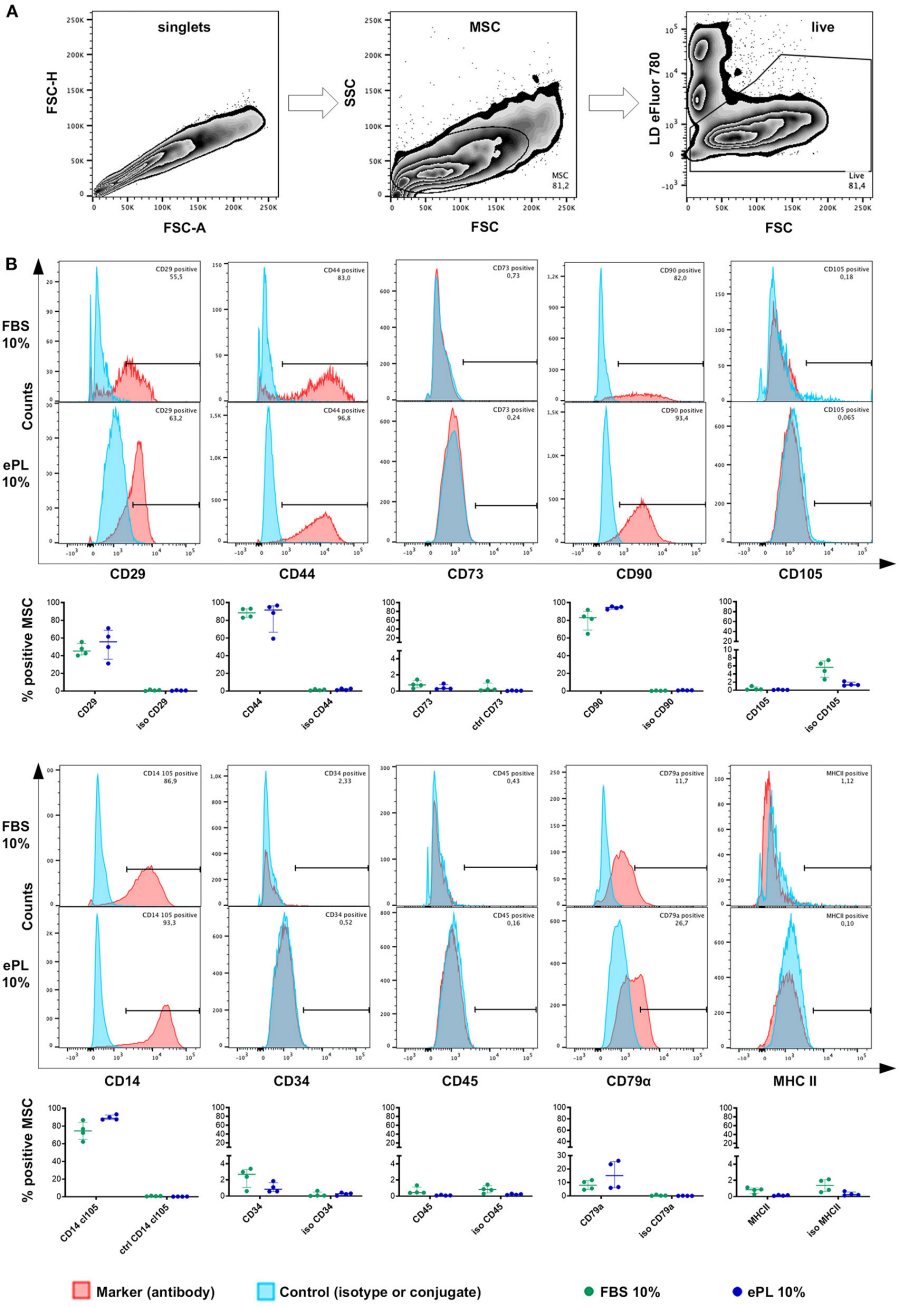
Flow cytometry analysis of equine mesenchymal stromal cells (MSC) cultured with 10% fetal bovine serum (FBS) or 10% equine platelet lysate (ePL) after cells were mechanically detached and stained for 10 different surface markers. **(A)** Gating strategy for live MSC after duplet exclusion. **(B)** Representative examples of overlaid histograms of the surface marker staining and the respective isotype or conjugate control for MSC from one donor, and frequencies of surface marker positive MSC as % of live MSC plotted as individual values; horizontal bars mark the median and interquartile ranges. Data were obtained from MSC from *n* = 4 donors.

MSC were positive for CD90, with the most consistent expression (≥92% CD90^+^ cells in all samples) in MSC cultured in 10% ePL. In addition, MSC expressed CD44 and CD29. However, MSC were largely negative for CD73 and CD105 in FBS and ePL media (Figure 6).

MSC hardly expressed CD34 (<4% in FBS medium and <2% in all ePL media), CD45 (<2% in all media), and MHC II (<2% in all media). Surprisingly, small subpopulations (up to 26.1% in 10% ePL medium) appeared to be positive for CD79α (Figure 6). Furthermore, MSC CD14 staining strongly depended on the antibody used (Supplementary File 2). Using the anti-equine CD14 antibody (clone 105), the majority of MSC appeared CD14-positive (≥62% CD14^+^ cells in 10% FBS medium and ≥87% CD14^+^ cells in 10% ePL medium) (Figure 6).

No statistically significant differences of the marker expressions were found between groups. Yet due to their low yield, MSC cultured in 5 or 2.5% ePL media could not be analyzed by flow cytometry in appropriate cell numbers and with appropriate controls. The attempted analysis suggested a similar but more inconsistent marker expression as in the other media, but this result is of preliminary character (data not shown).

#### MSC Trilineage Differentiation

**Figure 7:**
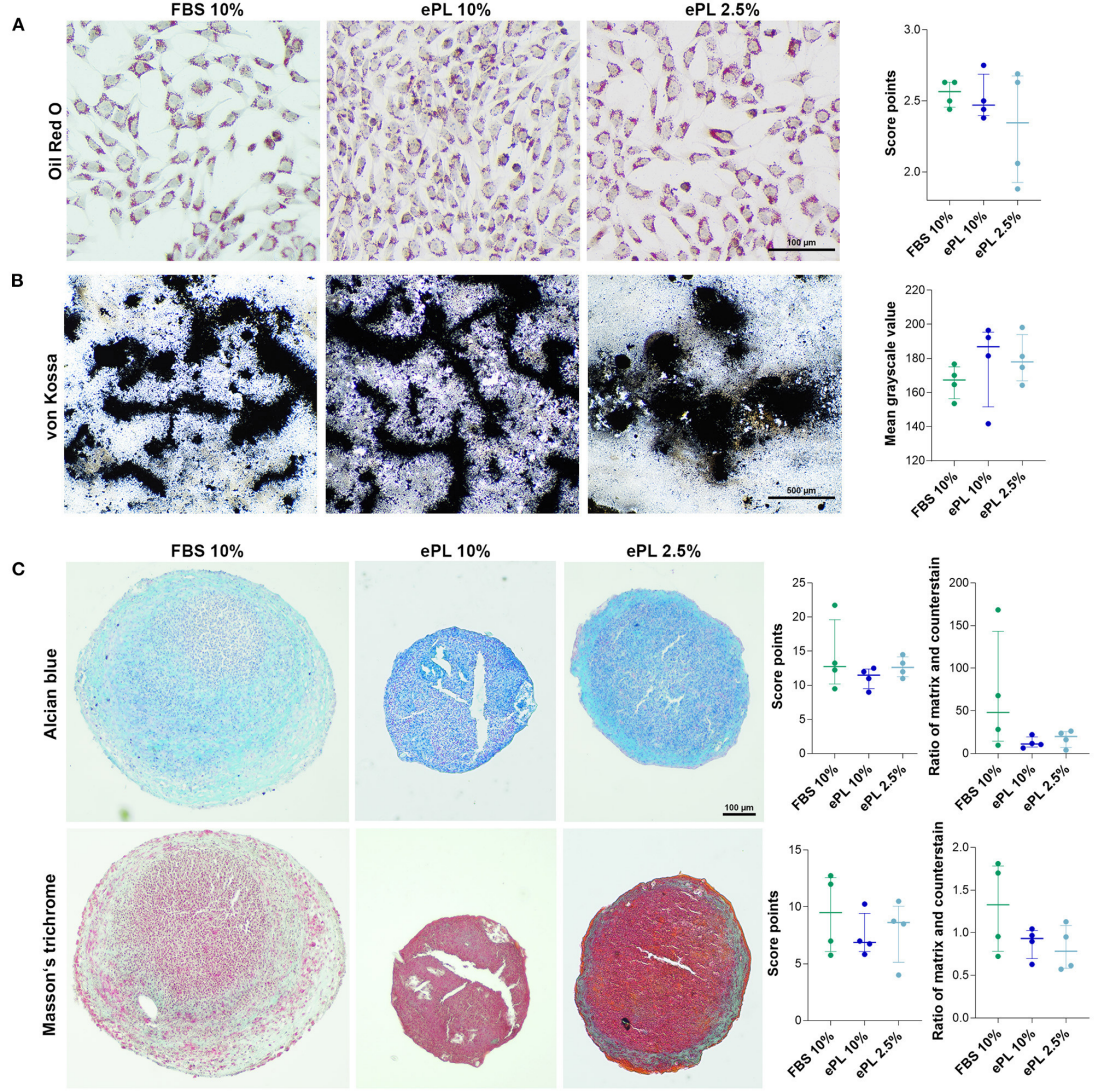
**(A)** Representative brightfield photomicrographs of equine mesenchymal stromal cells (MSC) after adipogenic **(A)**, osteogenic **(B)**, and chondrogenic **(C)** differentiation and corresponding data obtained by scoring **(A,C)** and image analysis using Fiji ImageJ **(B,C)**. MSC were cultured in the media indicated before differentiation was induced (FBS, fetal bovine serum; ePL, equine platelet lysate). The plots display the individual values, median, and interquartile ranges. Data were obtained from MSC from *n* = 4 donors.

Adipogenic differentiation was evident in all samples, with a high percentage of cells containing distinct lipid vacuoles. Similarly, all samples showed extensive extracellular mineral depositions after osteogenic differentiation. Chondrogenic differentiation appeared weaker in MSC expanded in 10% ePL, with the same trend being reflected by Alcian blue and Masson's trichrome stainings, yet these differences were not statistically significant (Figure 7).

## Discussion

Here, we propose a buffy coat-based protocol for equine PLT concentrate and ePL production, which could provide a basis for improved xeno-free cell culture media for equine MSC expansion. The ePL was prepared without the specific plateletpheresis equipment and with quality controls at different processing steps and can be reproduced in large scales.

PL is produced from PLT concentrate. The shelf life of human PLT concentrates prepared for transfusion medicine is limited to 5–7 days at 22 ± 2°C under permanent agitation to minimize the risk of bacterial growth (Corash, 2011; Burnouf et al., 2014). Even in well-organized blood transfusion services, 10–20% of the platelet concentrates produced have to be discarded because they were not transfused within the specified time (Schallmoser et al., 2020). Those expired PLT concentrates can be allocated for hPL production to avoid an additional donation from blood donors, because several studies showed no differences between fresh and stored PLT concentrates as a source for hPL (Bieback, 2013; Astori et al., 2016; Burnouf et al., 2016), thus a certain amount of PLT concentrate is always available for hPL production. However, in contrast to human medicine, PLT concentrates from transfusion services are not available in equine medicine and therefore, PLT concentrates must be prepared directly for ePL production.

Platelet concentrate can be obtained by three distinct procedures: Either from anticoagulated whole blood by the buffy coat-based or the platelet-rich plasma (PRP) method or directly by plateletpheresis. In the buffy coat method, PLT are harvested directly from the buffy coat after a so-called hard spin and subsequently separated from the white blood cells in a second soft spin. In the PRP method, a first soft spin centrifugation is followed by a second hard spin centrifugation, in order to first harvest the PLT together with the plasma supernatant and then pellet them, at which the second step might lead to reversible PLT aggregation (Gulliksson, 2012). In Europe, the buffy coat method and repeated freeze/thaw cycles represent the most commonly used techniques in human medicine, while the PRP method is not frequently used anymore (Gulliksson, 2012; Burnouf et al., 2016). In contrast, equine PLT concentrates have only been produced by the PRP method or plateletpheresis, with the PRP method performed in most studies, using small sample volumes only. In the present study, we propose the first buffy coat-based method to produce ePL from the donor horses' whole blood collected in commercial blood bags.

For the buffy coat as well as for the PRP method, centrifugation settings are critical for the correct separation of blood components. However, neither in human nor in equine medicine, protocols are standardized. In previous equine studies using the PRP method, the centrifugation settings for the first and second steps varied as detailed in Table 3. In human medicine, protocols are available for the buffy coat method, but they differ not only in the centrifugation settings (200–3,200 × *g* for the second centrifugation) but also in the PLT counts yielded in the concentrates (100–10,000 × 10^9^/L) (Burnouf et al., 2016).

**Table 3:** Preparation technique for equine platelet (PLT) concentrate and PLT lysate and resulting PLT counts in the platelet concentrates in previous publications from 2007 to 2019.

**References**	**First centrifugation**	**Second centrifugation**	**Final PLT concentration**	**Lysis**
Del Bue et al. (2007)	350 × *g*, 10 min	510 × *g*, 10 min	1,000–2,000 × 10^9^/L	Single freeze/thaw cycle + centrifugation (20,000 × *g*, 10 min)
Seo et al. (2013)	230 × *g*, 10 min, 10°C	900 × *g*, 15 min, 10°C	1,000 × 10^9^/L	Single freeze/thaw cycle + centrifugation (1,600 × *g*, 30 min, 10°C)
Russell and Koch (2016)	200 × *g*, 15 min	400 × *g*, 15 min	1,000 × 10^9^/L	Single freeze/thaw cycle + centrifugation (4,000 × *g*, 15 min)
Yaneselli et al. (2019)	200 × *g*, 10 min	1,500 × *g*, 20 min	591 × 10^9^/L	Single freeze/thaw cycle + centrifugation (1,600 × *g*, 30 min)
Bozorgmanesh et al. (2019)	1,000 × *g*, 5 min 45 s	2,000 × *g*, 8 min	713,333 × 10^9^/L	
Gilbertie et al. (2018)	250 × *g*, 15 min	1,500 × *g*, 15 min	1,226.38 × 10^9^/L	5 freeze/thaw cycles + centrifugation (20,000 × *g*, 20 min)
Sumner et al. (2017) and Naskou et al. (2018)	Plateletpheresis		357 ± 177 × 10^9^/L	2 freeze/thaw cycles + 3 centrifugation cycles (4,800 × *g*, 1.60 min and 2./3. 30 min) + centrifugation (3,485 × *g*, 10 min, 4°C)

Using the buffy coat-based method to produce equine PLT concentrate in the present study, we yielded a median PLT count of 484 × 10^9^/L. In previous equine studies, PLT counts ranged between 591 × 10^9^/L and 1,000–2,000 × 10^9^/L using the PRP method with multiple centrifugations (Del Bue et al., 2007; Seo et al., 2013; Russell and Koch, 2016; Gilbertie et al., 2018; Bozorgmanesh et al., 2019; Yaneselli et al., 2019) and 350 ± 106 × 10^9^/L to 357 ± 177 × 10^9^/L by plateletpheresis (Sumner et al., 2017; Naskou et al., 2018) (Table 3). Data regarding the buffy coat method for equine PLT concentrate production are not available for comparison. Nevertheless, the PLT concentration in the current study was in the same order of magnitude as described for the PRP method (Del Bue et al., 2007; Seo et al., 2013; Russell and Koch, 2016; Gilbertie et al., 2018; Bozorgmanesh et al., 2019; Yaneselli et al., 2019) and higher than the PLT concentrations yielded by plateletpheresis (Sumner et al., 2017; Naskou et al., 2018).

Besides PLT counts, WBC removal is a central aspect in PLT concentrate production and near-complete WBC depletion by filtration is recommended by the guidelines for transfusion procedures (Wildt-Eggen et al., 2001; European Committee (Partial Agreement) on Blood Transfusion, 2020; Schallmoser et al., 2020). Leukocytes can enhance PLT aggregation and thromboxane release, and, thus, promote further recruitment of activated PLT, as *in vitro* studies with human PLT have shown (Faraday et al., 2001; Hoareau et al., 2014). However, it should be differentiated between conventional intravenous PLT transfusion and other applications of PLT concentrates, such as local injections in regenerative therapies, where leukocyte removal is controversial (DeLong et al., 2012; Lana et al., 2019). With respect to concentrates for PL production as MSC culture supplement, it is unclear whether a reduction of leukocytes is necessary or whether leukocytes in the concentrate may be even useful in the sense of MSC priming. Yet, as this remains to be investigated, we aimed at a reduction of leukocytes in the present work. In a previous experiment (unpublished data), we had aimed to produce PLT concentrate by sedimentation of whole blood, followed by hard spin centrifugation of the supernatant similar to the PRP method. We had obtained higher PLT counts than with the buffy coat method presented here, but the leukocytes were also concentrated compared with the whole blood (unpublished data), which was in accordance with previous equine studies (Ionita et al., 2014; Bozorgmanesh et al., 2019). Furthermore, we did not succeed to deplete WBC using leukocyte reduction filters for the equine PLT concentrates. The filters were immediately clogged, which could be due to the different characteristics of equine PLT compared with human PLT (Roscher, 2019) or a stronger activation of the PLT in the filter and thus an increased surface activity. Therefore, to avoid increased leukocyte values, we used the buffy coat method for the preparation of ePL. In this line, it needs to be acknowledged that plateletpheresis, while requiring costly and specific equipment, has an even better outcome with respect to leukocyte reduction, with 0.15 × 10^9^ WBC/L (Sumner et al., 2017; Naskou et al., 2019).

Lysis of PLT concentrate releases growth factors and chemokines from the PLT. Repeated freeze/thaw cycles are mainly used for this purpose, but direct PLT activation by calcium chloride (CaCl_2_), sonication, or solvent/detergent treatment is also feasible (Astori et al., 2016; Burnouf et al., 2016; Schallmoser et al., 2020). In contrast to the method presented here, using three freeze/thaw cycles, other equine studies used only one (Del Bue et al., 2007; Seo et al., 2013; Russell and Koch, 2016; Yaneselli et al., 2019) or two freeze/thaw cycles and three centrifugation steps (Sumner et al., 2017; Naskou et al., 2018). During the last centrifugation after lysis, again different centrifugation forces were used (Table 3). A systematic analysis determining the optimal number and conditions of the freeze/thaw cycles and centrifugation settings is still pending.

In the present study, the growth factor concentrations in the PLT concentrates were in a similar range as in the ePL, suggesting that PLT lysis is already evident after one freezing step, which was necessary to store the concentrates until the ELISA analyses were performed. However, unexpectedly, we found a discrepancy between the median TGF-β1 concentration in the individual lysates of one horse and its concentration measured in the pooled ePL. This suggests that there is TGF-β1 activation during final processing steps, possibly leading to successively less activatable TGF-β1 over a short time. This issue should be further investigated and procedures or materials used improved. In previous studies, similar (PDGF-BB: 3.5 ng/ml and TGF-β1: 6.1 ng/ml) (Sumner et al., 2017) or higher (PDGF-BB: 5.2 ng/ml and TGF-β: 24.5 ng/ml) (Russell and Koch, 2016) growth factor concentrations were found in ePL prepared from plateletpheresis- or PRP method-derived concentrates, respectively. Furthermore, corresponding to previous data (Russell and Koch, 2016), we could not detect PDGF-BB in FBS. Yet, the concentrations of growth factors measured may not only vary depending on the PL preparation process, but they are also affected by the assay specificity (Doucet et al., 2005; Textor and Tablin, 2012; Sumner et al., 2017), thus direct comparisons between studies or samples from different species are not entirely conclusive.

It was previously reported that breed, sex, and age of the donors can influence the cellular and growth factor profile of equine PLT products. Female horses and horses aged <5 years showed significantly higher concentrations of PDGF-BB and TGF-β1 in blood products. For PDGF, this was also observed in pony breeds (Giraldo et al., 2013). These findings were partially reproduced in our study, in which the PLT and growth factor concentrations in the PLT concentrates and lysates were negatively correlated with the age of the horses. To overcome the individual variations, pooling of the ePL from a large number of animals is beneficial for producing uniform batches. Nevertheless, it remains recommendable to harvest the blood samples from young donor horses in uniform cohorts in order to obtain PLT concentrate and ePL of high quality.

We further compared the obtained ePL with FBS, in terms of their potential to support equine adipose-derived MSC expansion. PL is an attractive alternative to FBS, which could solve several issues related to FBS. First, it is an allogeneic product, which reduces the possibility of recipient immune reaction, and secondly, harvesting the materials for ePL production is ethically less critical. However, given that the use of MSC and other cell types in large animal models and veterinary medicine will further increase, ePL would be required in large quantities for MSC expansion. Furthermore, producing large batches of ePL according to standardized protocols would support consistent cell culture conditions. In this respect, it is a major advantage that horses tolerate regular blood collection of large amounts up to 8 L (16 ml/kg bodyweight) very well, and there are no adverse effects in the hematological variables (Malikides et al., 2000).

When analyzing the MSC proliferation kinetics, the most consistent viability and proliferation was observed in medium supplemented with 10% ePL. There were no major differences between 10% ePL MSC and 10% FBS MSC with respect to the proliferation parameters assessed. This supports the notion that ePL can be used as a replacement of FBS for equine MSC expansion. Our results are in agreement with the findings of two other studies, one using the PRP method (Seo et al., 2013) and the other using plateletpheresis (Naskou et al., 2018) to produce PLT concentrate and ePL. Furthermore, two other equine studies showed a dose-dependent proliferation of MSC in ePL medium (Del Bue et al., 2007; Russell and Koch, 2016). Yet only in one study, using supplementation with 20% ePL, MSC proliferation was actually increased as compared with FBS (Yaneselli et al., 2019). In comparison with these results, it is well-documented that hPL supplementation increases human bone marrow- and adipose-derived MSC proliferation as compared with FBS (Doucet et al., 2005; Blande et al., 2009; Cholewa et al., 2011; Becherucci et al., 2018). As already suggested earlier (Naskou et al., 2018), we also tested different concentrations of ePL for equine MSC cultivation. However, the proliferation was highly variable in medium supplemented with 5% ePL and insufficient in medium supplemented with 2.5% ePL. This is again in conflict with the findings in human medicine, where basal media supplemented with 5% hPL resulted in a significant increase in the proliferation rates of human MSCs compared with supplementation with FBS (Griffiths et al., 2013). Here, species-specific differences become evident. In contrast to an optimal growth factor content in 5% hPL for improved expansion of human MSC, the growth factor content in a medium supplemented with 5 or 2.5% ePL was not sufficient to support consistent equine MSC expansion. The different PLT concentrations in human and equine whole blood are a probable reason for this. The reference range for PLT counts in humans is 150–450 × 10^9^/L (Burnouf et al., 2016), but only 94–232 × 10^9^/L in horses (Stokol, 2020). Based on the resulting higher PLT count in the human PLT concentrates, it is not surprising that the growth factor concentrations reported for hPL (Klatte-Schulz et al., 2018) appear higher than in ePL, which may explain that less hPL is needed to support cell growth.

Human MSC characterization, as recommended by the ISCT, includes the evaluation of the positive marker antigens CD73, CD90, and CD105 and the exclusion marker antigens CD14, CD34, CD45, CD79α, and HLA-DR (Dominici et al., 2006). For equine MSC, it was suggested to further include CD29 and CD44 as inclusion markers (Paebst et al., 2014; Hillmann et al., 2016). Until today, the scarcity of reliable commercially available monoclonal antibodies specific for equine cells is a major drawback (Schauwer et al., 2011). The current analysis revealed that the immunophenotype of MSC cultured in media supplemented with FBS or ePL is similar. There were no significant differences in the expression of positive markers CD29, CD44, CD90, CD73, and CD105, while CD73 and CD105 were largely negative in all media. Furthermore, the MSC did not express the exclusion markers CD34, CD45, and MHC II. These observations are widely comparable with other equine and human studies, where similar expression of surface markers was observed with different media (Doucet et al., 2005; Horn et al., 2010; Mojica-Henshaw et al., 2013; Yaneselli et al., 2019). Lack of CD73 in equine MSC is also in accordance with most previous equine studies (Ranera et al., 2011; Schauwer et al., 2012; Paebst et al., 2014; Hillmann et al., 2016) while CD105 has shown both high (Braun et al., 2010) or minimal or variable expressions in previous studies (Ranera et al., 2011; Schauwer et al., 2012; Paebst et al., 2014). However, in one previous study, a CD45^+^ population was observed in MSC cultured with FBS as well as with ePL, and CD90 expression was found in a higher percentage of MSC cultured with FBS compared with ePL (Naskou et al., 2018). In contrast, we observed the most consistent expression of CD90 in MSC cultured with 10% ePL media and expression of CD45 was almost undetectable in all media. Furthermore, we made an interesting observation of CD14 detection on the majority of MSC when using an equine-specific antibody for CD14 (Kabithe et al., 2010; Wagner et al., 2013; Bonelli et al., 2017; Schnabel et al., 2019; Larson et al., 2020; Patel et al., 2020). According to the ISCT, CD14 should not be expressed on human MSC (Dominici et al., 2006) as it is a glycolipid anchored membrane glycoprotein expressed on monocytes and macrophages (Tesfaigzi, 2006; Braun et al., 2010) and a leukocyte-secreted molecule in response to LPS stimulation and inflammation, as demonstrated in horses (Wagner et al., 2013). Previously, we described equine MSC as CD14 negative (Paebst et al., 2014). These analyses were based on an anti-human CD14 antibody (clone 134620), which stains putative monocytes in equine peripheral mononuclear blood cells but not equine MSC (Paebst et al., 2014) (Supplementary File 2), but its exact specificity for equine CD14 has not been shown. In the current work, we used an equine-specific antibody for the flow cytometric analysis (clone 105, Kabithe et al., 2010), which discriminates the monocyte population in peripheral blood mononuclear cells more clearly (Supplementary File 2). While our current finding of equine MSC staining positive for CD14 is in accordance with previous studies (Braun et al., 2010; Hackett et al., 2011), it remains to be clarified if the cells actually express the whole functional CD14 molecule or only part of it, or if the MSC bind soluble CD14 present in FBS and ePL. On human MSC, cross-reactive epitopes have been observed, despite absence of the complete CD14 molecule (Pilz et al., 2011). The details and relevance of our finding of CD14^+^ equine MSC here will be subject of further research.

We finally verified MSC *in vitro* trilineage differentiation potential. In accordance with previous studies, the MSC expanded in ePL showed no significant differences in osteogenic and adipogenic differentiation (Doucet et al., 2005; Schallmoser et al., 2007; Ben Azouna et al., 2012; Naskou et al., 2018). Interestingly, MSC expanded in medium supplemented with 2.5% ePL differentiated into both adipocytes and osteocytes, although their proliferation had not been satisfying in this medium. In addition, we obtained unexpected results in chondrogenic differentiation, which was weaker in MSC expanded in 10% ePL medium than when expanded in FBS or 2.5% ePL medium. In contrast, others reported that chondrogenic differentiation was improved in equine and human MSC cultured with ePL (Mishra et al., 2009; Shih et al., 2011; Gottipamula et al., 2012; Naskou et al., 2018). It is widely discussed in the literature that MSC express the chondrogenic markers aggrecan and Sox 9 and extracellular cartilage matrix depending on the amount of (platelet-derived) growth factors (Mishra et al., 2009; Prins et al., 2009; Shih et al., 2011; Rubio-Azpeitia and Andia, 2014). In this context, TGF-β appeared to be crucial for chondrogenic differentiation (Grimaud et al., 2002; Chapman et al., 2020). A possible and pragmatic explanation for the discrepancy observed here could be that MSC expanded in 10% ePL medium would have needed more extensive washing steps to remove the ePL completely before being transferred to the chondrogenic differentiation medium.

The current study provides the first evaluation of a buffy coat-based protocol for ePL production. The method presented has several advantages over previously described protocols for equine PLT concentrate and ePL production and complies much better with the current state of the art in human medicine. Therefore, it can serve as a basis to improve the culture of equine MSC and other equine cell types. MSC characterization in the present study was limited in that only four MSC donor horses were investigated, and because so far, we only investigated basic properties of MSC but not their functionality in clinically relevant settings. Based on the herein obtained results, it appears highly promising to continue with the functional characterization of equine MSC in conjunction with their culture in ePL.

## Data Availability Statement

The raw data supporting the conclusions of this article will be made available by the authors, without undue reservation.

## Ethics Statement

The animal study was reviewed and approved by Regional council Giessen, Germany. Written informed consent was obtained from the owners for the participation of their animals in this study.

## Author Contributions

AH: conception of the study and complete experimental design (together with JB), sample acquisition, blood processing, and MSC culture experiments, sample and data analysis, data interpretation and drafting of the manuscript (together with JB). HL: substantial contribution to the experimental design, help with blood processing, analysis, and data interpretation (blood processing). SA: substantial contribution to the experimental design, microbiological analysis, and data interpretation (microbiology). NB: substantial contribution to the experimental design and data interpretation (blood processing). MM: contribution to the experimental design, MSC culture experiments, sample analysis (MSC culture and differentiation). JM: contribution to the experimental design, help with sample acquisition, histology, sample analysis (blood processing and MSC differentiation). VP: contribution to the experimental design, help with blood processing, and hematological sample analyses (blood processing). CS: substantial contribution to the experimental design, sample and data analysis, data interpretation, and drafting of the manuscript (flow cytometry experiments). JB: conception of the study and complete experimental design (together with AH), help with MSC culture experiments, data analysis, data interpretation and drafting of the manuscript (together with AH). All authors have critically revised the manuscript for important intellectual content and approved the publication of its content.

## Conflict of Interest

The authors declare that the research was conducted in the absence of any commercial or financial relationships that could be construed as a potential conflict of interest.
