# Supplementary material for: Scalable Production of Equine Platelet Lysate for Multipotent Mesenchymal Stromal Cell Culture
Source: Front Bioeng Biotechnol. 2021 Jan 21;8:613621. doi: 10.3389/fbioe.2020.613621 (PMC7859354; doi:10.3389/fbioe.2020.613621)
Supplement: Supplementary file 1 [file Data_Sheet_1.pdf]

Hagen et al., Supplementary file 1:
Adapted Grogan score for semiquantitative analysis of chondrogenic differentiation

**Score categories**	**Score**
**A) Uniformity and lightblue*[1]/ blueish*[2] of Alcian blue/ Masson´s Trichrome stain**	
No stain	0
Weak staining of poorly formed matrix	1
Moderately even staining	2
Even strong stain	3
**B) Distance between cells/ amount of matrix accumulated**	
High cell densities with no matrix in between (no spacing between cells)	0
High cell densities with little matrix in between (cells < 1 cell–size apart)	1
Moderate cell density with matrix (cells approx. 1 cell-size apart)	2
Low cell density with moderate distance between cells (> 1 cell) and an extensive matrix	3
**C) Cell morphologies represented**	
Condensed/ necrotic/ pycnotic bodies	0
Spindle/ fibrous	1
Mixed spindle/ fibrous with rounded chondrogenic morphology	2
Majority rounded/ chondrogenic	3
**D) % of differentiated areas of the pellet in field of view (10x magnification)**	
0 - 5%	0
5 – 30%	1
30 – 60%	2
60 – 100%	3
[1]= Alcian blue staining<br>[2]= Masson´s Trichrome staining	
