# Supplementary material for: Scalable Production of Equine Platelet Lysate for Multipotent Mesenchymal Stromal Cell Culture
Source: Front Bioeng Biotechnol. 2021 Jan 21;8:613621. doi: 10.3389/fbioe.2020.613621 (PMC7859354; doi:10.3389/fbioe.2020.613621)

Hagen et al., Supplementary file 2:
Flow cytometric analysis of CD14 expression on equine peripheral blood mononuclear cells (PBMC) and equine mesenchymal stromal cells (MSC) using the antibody clones 105 and 134620

Equine cryopreserved PBMC after 2 h medium incubation or mechanically detached MSC cultured with 10% fetal bovine serum (FBS) or 10% equine platelet lysate (ePL) were stained for surface CD14 and analyzed by flow cytometry. Either biotinylated anti-equine CD14 clone 105 (Kabithe et al., 2010) was used (followed by Streptavidin-APC) or anti-human CD14 clone 13460 (Paebst et al., 2013) (directly conjugated with APC). The gating strategies are illustrated for each cell type by zebra plots; large mononuclear cells (MNC) in PBMC comprise mainly monocytes and few large lymphocytes (A). Examples of overlaid histograms of the surface marker staining and the respective conjugate or isotype control are shown (B). The frequencies of surface marker positive MSC as % of live MSC are plotted, horizontal bars mark the median and interquartile range of n=4 (C).

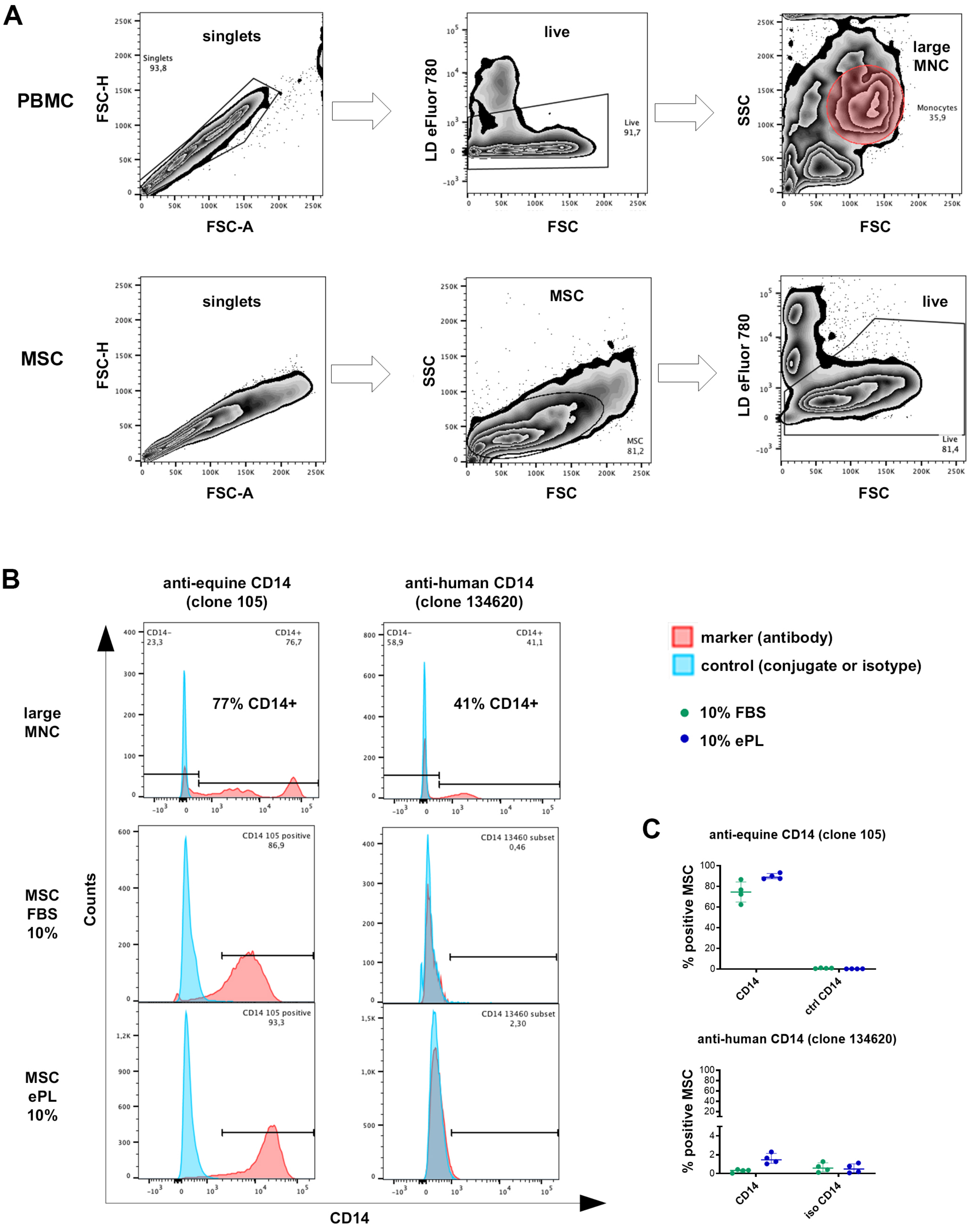

Supplement: Supplementary file 2 [file Data_Sheet_2.PDF]
